# Intra-unit-cell magnetic correlations near optimal doping in YBa_2_Cu_3_O_6.85_

**DOI:** 10.1038/ncomms8705

**Published:** 2015-07-03

**Authors:** L. Mangin-Thro, Y. Sidis, A. Wildes, P. Bourges

**Affiliations:** 1 Laboratoire Léon Brillouin, IRAMIS/LLB, UMR12, CEA-CNRS, CEA-Saclay, Gif sur Yvette 91191, France; 2 Institut Laue-Langevin, 71 avenue des martyrs, Grenoble 38000, France

## Abstract

The pseudo-gap phenomenon in copper oxide superconductors is central to any description of these materials as it prefigures the superconducting state itself. A magnetic intra-unit-cell order was found to occur just at the pseudo-gap temperature in four cuprate high-*T*_c_ superconducting families. Here we present polarized neutron-scattering measurements of nearly optimally doped YBa_2_Cu_3_O_6.85_, carried out on two different spectrometers, that reveal several features. The intra-unit-cell order consists of finite-sized planar domains that are very weakly correlated along the *c* axis. At high temperature, only the out-of-plane magnetic components correlate, indicating a strong Ising anisotropy. An aditional in-plane response develops at low temperature, giving rise to an apparent tilt of the magnetic moment. The discovery of these two regimes puts stringent constraints, which are tightly bound to the pseudo-gap physics, on the intrinsic nature of intra-unit-cell order.

## Abstract (web summary)

The pseudo-gap phenomenon is central to the description of high-*T*_c_ superconductivity in copper oxides. Here, the authors investigate nearly optimally doped YBCO using polarized neutron scattering to characterize intra-unit-cell magnetic correlations in relation with the pseudo-gap temperature.

## Graphical abstract

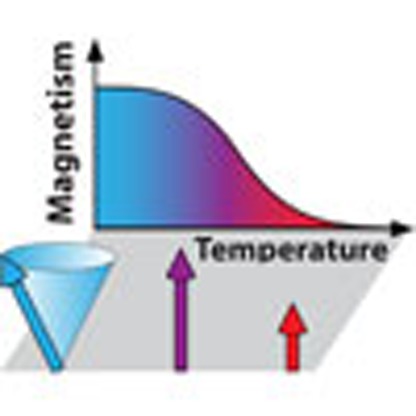

Understanding high-temperature superconductivity requires a prior knowledge of the nature of the enigmatic pseudo-gap metallic state^1^, out of which the superconducting state condenses. In addition to the electronic orders involving charge degrees of freedom that were recently reported inside the pseudo-gap state^2^,^3^,^4^, polarized neutron-scattering experiments reported the existence of a magnetic intra-unit-cell (IUC) order in four different cuprate families^5^,^6^,^7^,^8^,^9^,^10^,^11^,^12^,^13^,^14^. The IUC magnetic order breaks time-reversal symmetry but preserves the lattice translational symmetry (LTS). The IUC order develops below a temperature, *T*_mag_, that matches the pseudo-gap temperature, T*, as defined by resistivity measurements^15^,^16^, and where recent resonant ultrasound spectroscopy measurements in YBa_2_Cu_3_O_6__+*x*_ (YBCO) show that the pseudo-gap state is a true symmetry breaking phase^17^. While bulk neutron-scattering measurements provide unambiguous evidence for an IUC magnetic order, such an order was not detected by local magnetic probe measurements^9^,^18^,^19^. The conundrum could be understood in terms of slowly fluctuating magnetic domains with finite size^20^, although their existence has not yet been established.

The observed IUC magnetism can be described simply as a *q*=0 antiferromagnetic state as it keeps the LTS, but requires an internal staggered magnetic pattern inside each unit cell. Such a state is qualitatively consistent with the loop current (LC) model for the pseudo-gap^20^,^21^. While out-of-plane magnetic moments should be produced by the planar confinement of LC, the observed magnetic moments also display an unexpected in-plane component, giving rise to a tilt of ∼45° with respect to *c* axis^5^,^9^,^11^,^12^ at low temperature. Several attempts to explain this observation were proposed: an additional spin response through spin-orbit coupling^22^; a geometrical tilt of moment through delocalization of the LC over CuO octahedra or pyramids^23^,^24^; and a superposition of LC states through quantum effects^25^. Beside the LC model, electronic phases that break time-reversal symmetry, but preserve LTS, were proposed to pre-empt either a pair density wave state^26^ or a composite charge density wave (CDW) state^27^. Alternative scenarios consider an IUC order based on a nematic-like state with either spin^5^ or orbital^28^ moments on oxygens, or entangled spin and orbital degrees of freedom within an arrangement of magneto-electric multipoles^29^.

More experimental information is required on the size and lifetime of the magnetic domains and exact orientation of the magnetic moment to make progress on the understanding of the intrinsic nature of the IUC magnetic order and its interplay with the pseudo-gap physics. This can be achieved from studying compositions around optimal doping. Indeed, a fast decay of the magnetic intensity was reported from underdoped to optimally doped samples of Bi_2_Sr_2_CaCu_2_O_8+*δ*_ (ref. 14). The magnetic intensity is proportional to *T*_mag_ in the underdoped regime, whereas this scaling suddenly breaks down on approaching optimal doping. These effects could actually be triggered by a redistribution of the magnetic scattering in momentum space, namely, a shortening of the magnetic correlation length on increasing hole doping.

We here report polarized neutron measurements on a YBa_2_Cu_3_O_6.85_ (*T*_c_=89 K) twinned single crystal, previously used to study the spin dynamics^30^,^31^, extending the study of the IUC order at a higher hole doping *p*=0.15 near optimal doping. We find short-range IUC correlations with finite-sized planar domains of ∼*ξ*_*a*_ ∼75 Å at 100 K, which are very weakly correlated along the *c* axis. The magnetic intensity appears at high temperature with a net increase below *T*_mag_ ∼200 K. Polarization analysis reveals a strong Ising anisotropy at high temperature as originally predicted in the LC model^21^. Below *T*_mag_, transverse components correlate as well, leading to an apparent tilt of the magnetic moment at lower temperature. These different temperature dependencies of both in-plane and out-of-plane components of the magnetic moment suggest a distinct origin of each component, providing information on the nature of the tilt.

## Results

### IUC magnetism near optimal doping

**Figure 1:**
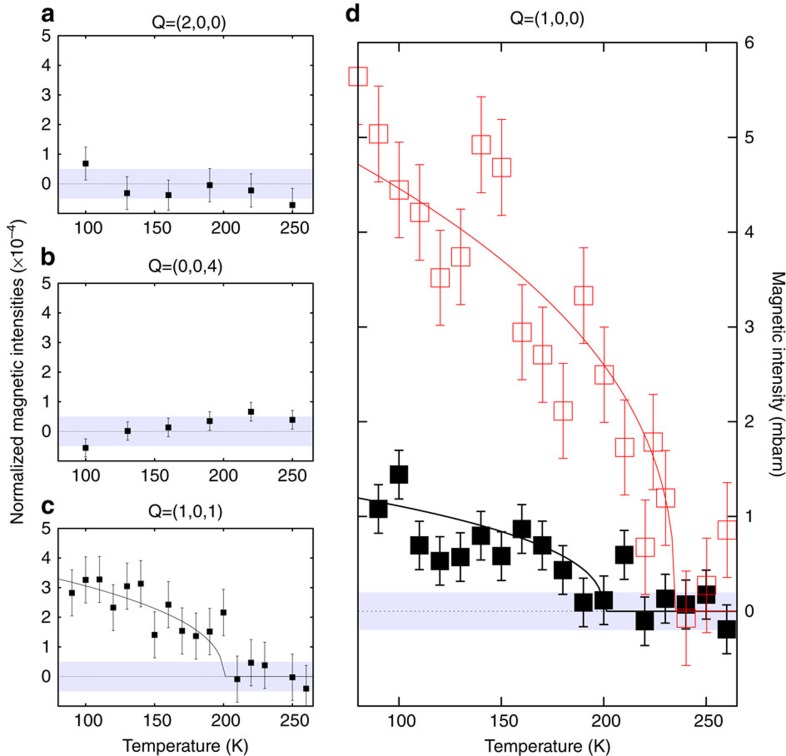
Magnetic intensity at Bragg peak positions. 4F1 data: temperature dependencies of normalized magnetic intensity (=*I*_mag_/*I*_NSF_, where *I*_mag_ is the expected magnetic intensity of the IUC order) measured at the wave vector (**a**) **Q**=(2,0,0) and (**b**) **Q**=(0,0,4) as references, and at (**c**,**d**) **Q**=(1,0,*L*) for the IUC magnetic order study in the **P**||**Q** configuration. (**c**) *L*=1, (**d**) *L*=0. The data for **Q**=(1,0,0) (YBa_2_Cu_3_O_6.85_, full black squares) have been calibrated in absolute units using the intensity of the Bragg peak **Q**=(0,0,4) (ref. 5), and further compared with the YBa_2_Cu_3_O_6.6_ study (red empty squares)^11^. Intensity error bars are statistical error bars calculated by the square root of the measured intensity.

At lower doping, evidence has been found for IUC magnetic order in YBCO by plotting normalized magnetic intensity, that is, the ratio of the spin flip (SF) to the non-SF (NSF) intensities at various Bragg reflections^5^,^11^,^13^. The IUC magnetic intensity occurs for Bragg reflections such as **Q**=(1,0,*L*) with *L*=integer, whereas it is absent for **Q**=(0,0,*L*) or **Q**=(2,0,*L*) (refs 9, 10). We show in Fig. 1 the results from the spectrometer 4F1 (see Methods section) as a function of the temperature, for a polarization **P**||**Q**, where the magnetic scattering is exclusively SF^9^. The raw data are shown in Supplementary Fig. 1 and the detailed analysis is given in Supplementary Note 1 following a method previously discussed^13^,^14^. In Fig. 1a,b, we plot the normalized magnetic intensity obtained at **Q**=(2,0,0) and **Q**=(0,0,4), where a magnetic signal (if any) is beyond the threshold of detection (<5 × 10^−5^). This serves as a reference calibration for the neutron flipping ratio.

Figure 1c shows the normalized magnetic intensity for the Bragg reflections **Q**=(1,0,1). Starting from zero scattering at high temperature, a magnetic signal appears below *T*_mag_ ∼200±20 K. Our measurements first made on the Bragg reflection **Q**=(1,0,1) are confirmed by those on **Q**=(1,0,0) (Fig. 1d), where the data are further compared with a previous study on YBa_2_Cu_3_O_6.6_ (ref. 11). Such a value for *T*_mag_ agrees with the recent accurate determination of T* from resistivity^32^. The magnetic signal displays a characteristic *T* dependence that can be fitted by (1−*T*/*T*_mag_)^2*β*^ with 2*β*=0.4±0.17, a value similar to what was found in YBa_2_Cu_3_O_6.6_ (ref. 11). Next, one can convert this magnetic amplitude in absolute units following a method described in previous studies^5^,^9^. The amplitude of the magnetic intensity, *I*_mag_, for both **Q**=(1,0,*L*) is ∼3 × 10^−4^ of the nuclear intensity, giving *I*_mag_ ∼1.2 mbarns per formula unit for **Q**=(1,0,0) and *I*_mag_ ∼0.4 mbarns per formula unit for **Q**=(1,0,1).

### Short-range magnetic order

**Figure 2:**
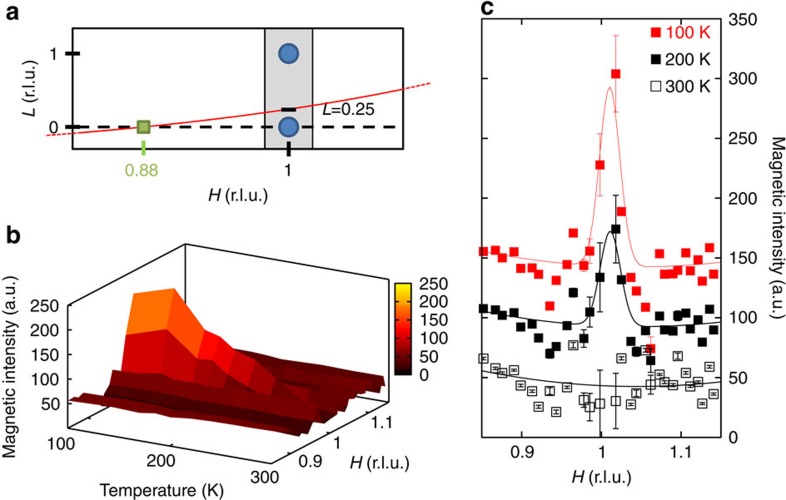
In-plane momentum dependence of the magnetic correlations. (**a**) Schematic view of the (*H*,0,*L*) reciprocal space probed on D7 with the measured trajectory going through (1,0,0.25) in red. The grey area represents the location of the magnetic intensity. r.l.u., reduced lattice units. The green spot represents the location where one observes diffuse scattering around *H*=0.88 shown in Fig. 4. (**b**) 3D plot of the magnetic intensity in the SF channel around **Q**=(1,0,0.25) as a function of temperature and wave vector. (**c**) Cuts of the 3D map along (*H*,0,0.25) at three temperatures: 100 K (red squares), 200 K (brown squares) and 300 K (black empty squares). Data obtained in channels *X* and *Z* are here averaged. Intensity error bars are statistical error bars calculated by the square root of the measured intensity.

To gain a new insight of the IUC magnetism, we next carried out a polarized neutron experiment using the multidetector diffractometer D7 covering a wide range of scattering angle (see Methods section and Supplementary Note 2). A trajectory in the scattering plane (as shown in Fig. 2a) is simultaneously measured for a given sample rocking angle. A survey of a few trajectories in the **Q**=(*H*,0,*L*) scattering plane showed the presence of magnetic scattering around *H*=1 not only for the Bragg reflection (1,0,0) but also for non-integer *L*. In particular, the trajectory across **Q**=(1,0,0.25) (Fig. 2a) showed that the magnetic intensity is still large, whereas the nuclear Bragg scattering is significantly reduced (by about one order of magnitude), improving noticeably the ability to observe the magnetic scattering compared with the Bragg position at **Q**=(1,0,0). The magnetic scattering is thus short range along a rod **Q**=(1,0,*L*) with an extension along c*, in contrast to more underdoped YBCO samples^11^. Similar short-range magnetic fluctuations around *H*=1 were also reported in La_1.92_Sr_0.08_CuO_4_ (ref. 7). One can therefore extract the in-plane correlations length *ξ*_*a*_ (which is equivalent to *ξ*_*b*_ as our sample is twinned) as well as the correlations length along c*.

In Fig. 2b,c, measurements along **Q**=(*H*,0,0.25) showed that the magnetic signal increases on cooling. At high temperature (300 K), the magnetic signal is assumed to be featureless, whereas at low temperature (100 K) there is a net enhancement of the magnetic intensity centred at *H*=1. From the *H*-dependences of Fig. 2c, one can extract the *q*-width by fitting a Gaussian, Δ*H*=0.025±0.004 r.l.u. (reduced lattice units) (full-width at half-maximum). We observed no noticeable evolution of that *q*-width with temperature. Actually, Δ*H* is slightly broader than the resolution Δ*H*_res_=0.0195±0.001 r.l.u., suggesting short-range correlations. One can extract a finite planar magnetic correlation length, *ξ*_*a*_=*a*/(*π.*Δ*H*_*i*_), where *a*=3.85 Å is the in-plane lattice parameter and 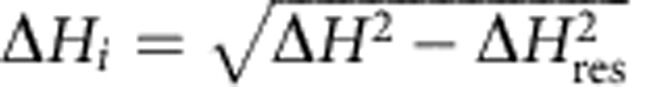 is obtained by deconvolution from the resolution: *ξ*_*a*_ ∼20*a* ∼75 Å. Such a correlation length cannot be determined on 4F1 due to its poorer *H*-resolution (see Methods section).

**Figure 3:**
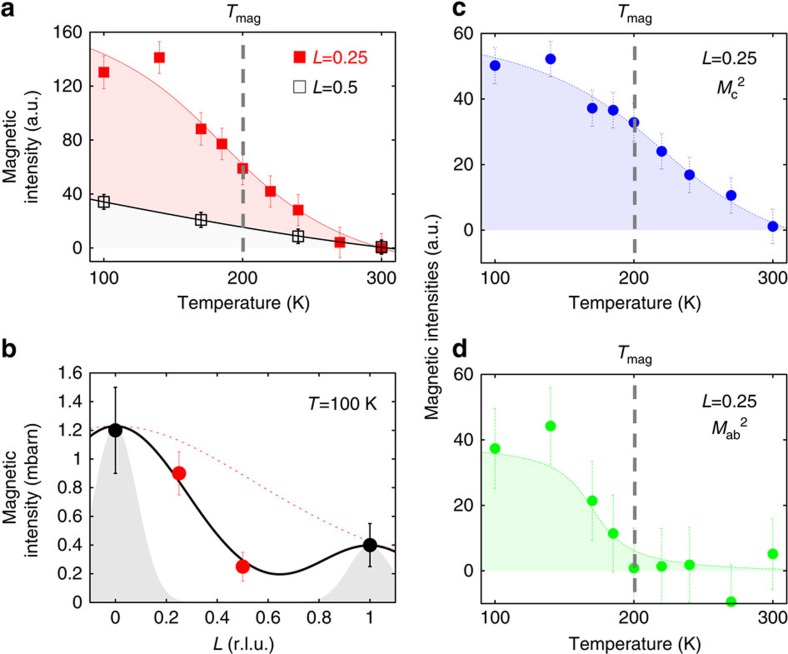
Temperature and out-of-plane momentum dependence of the magnetic correlations. (**a**) Temperature dependences of the magnetic intensity, 
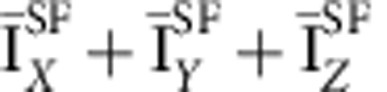
 (background subtracted) for two *L*. (**b**) Magnetic intensity in absolute units from 4F1 (black symbols) and D7 (red symbols) versus *L* at 100 K. The dashed areas represent Bragg peaks with the *L*-resolution of the spectrometers. The red dotted line corresponds to the universal decay of the magnetic intensity along the magnetic rod **Q**=(1,0,*L*) found in all cuprates^8^,^14^. Temperature dependence of the magnetic components for *L*=0.25: (**c**) the out-of-plane component 
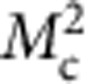
 and (**d**) the in-plane component 
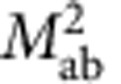
 (Supplementary Note 4). Solid lines are guides to the eye. Intensity error bars are statistical error bars calculated by the square root of the measured intensity.

Next, to improve the statistics of signal, we proceeded to a partial integration of the scattering intensities over 10 detectors (Supplementary Note 3). This averaged intensity, *I*_av_, is reported as a function of temperature (Fig. 3a) along the magnetic rod **Q**=(1,0,*L*) for two *L* values (*L*=0.25 and 0.5) off the Bragg position. Here we have summed up the cross-sections of the three different neutron polarizations in the SF channel 
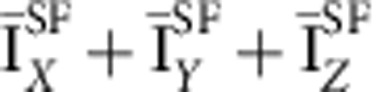
. Using that method, weak magnetic correlation can be detected even at high temperature above *T*_mag_ ∼200 K. As the sample is cooled, the comparison of the magnetic intensity at both *L* values in Fig. 3a shows that the magnetic intensities at first grow similarly at both *L* and then more rapidly for *L*=0.25. That behaviour suggests a gradual redistribution of the magnetic intensity along the (1,0,*L*) rod towards the magnetic Bragg positions, pointing towards a two-dimensional (2D) to three-dimensional (3D) crossover of the magnetic correlations with temperature.

By converting the magnetic intensity to absolute units, one can compare data from both 4F1 and D7 (Fig. 3b): the scattering is broader along c* than the *L*-resolution width given by Δ*L*_res_=0.2 r.l.u. (see Methods section). Using a Gaussian fit, one estimates the full-width at half-maximum of the magnetic signal along (001), Δ*L*=0.65±0.05 r.l.u. at 100 K, out of which one can extract the correlation length along c*, *ξ*_*c*_=*c*/(*π.*Δ*L*_*i*_), where 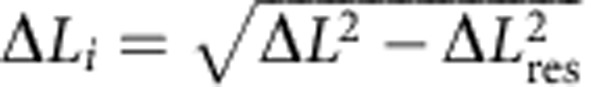 is obtained by deconvolution from the resolution. It gives a very short correlation length along c*, *ξ*_*c*_ ∼0.5*c* at *T*=100 K. That observed *L*-dependence is intermediate between two limiting cases: 2D correlations (dotted line in Fig. 3b) as it is observed in La_1.92_Sr_0.08_CuO_4_ (ref. 7) and well-defined 3D ordering (shaded Gaussian peaks in Fig. 3b) in more underdoped YBCO where *ξ*_*c*_>7*c* (refs 9, 11).

### Magnetic components

One can also use the temperature dependence of the averaged intensity to estimate the directional components of the magnetic moment, *M*=(*M*_a_,*M*_b_,*M*_c_). For a twinned sample, both directions (1,0) and (0,1) are equivalent, and one can define 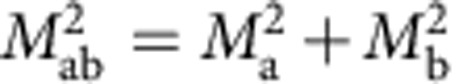 as the in-plane component and 
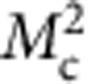
 as the out-of-plane component^9^. As described in the Supplementary Note 4, the cross-section in the SF channel 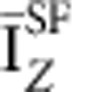 essentially corresponds to the out-of-plane magnetic component, 
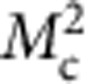
, in the limit *q*_l_=(2*πL*/*c*)/|Q| → 0 (as it is the case for *L*=0.25), and conversely the quantity 
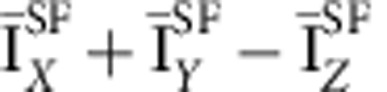
 directly probes the in-plane component 
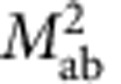
. In Fig. 3c,d, we report the temperature evolution of the out-of-plane component 
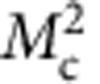
 and the in-plane component 
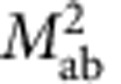
. Interestingly, both cross-sections exhibit noticeably distinct temperature dependences. At high temperature, the in-plane component vanishes, meaning that the correlated magnetic fluctuations <*M*^2^> essentially correspond to the out-of-plane component. That strong Ising character (along c*) remains down to *T*_mag_. Below *T*_mag_, the in-plane component also increases. As *I*_av_ ∝ *I*_0_/*ξ*_*a*_ (where *I*_0_ is the magnetic peak intensity), it indicates that both magnetic components have a distinct temperature-dependent evolution in their amplitudes and/or in their correlation lengths.

Following the definition of the magnetic moment, the ratio between *M*_ab_ and *M*_c_ at 100 K can be used to define an apparent tilt angle, *θ*, of the magnetic moment with respect to the c* axis as tan(*θ*)=*M*_ab_/*M*_c_ (ref. 9) from Fig. 3c,d, tan(*θ*) ∼0.84, giving *θ*∼40°±9° for *L*=0.25. As shown by Fig. 3b, the magnetic correlations at *L*=0.25 belong to the same peak as *L*=0, so that tilt value holds for *L*=0 as well. Actually, a similar value for the tilt for measurements at *L*=1 has been reported previously for more underdoped YBCO samples^5^,^9^,^11^.

**Figure 4:**
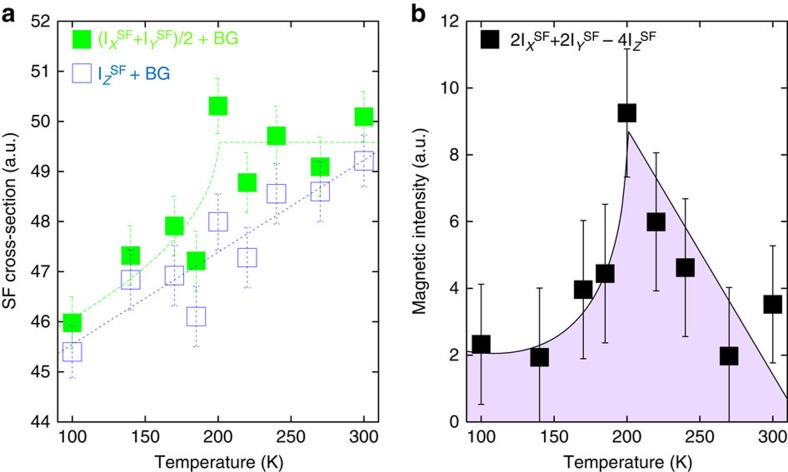
Temperature dependence of the magnetic diffuse scattering. (**a**) Temperature dependencies of the SF cross-sections around **Q** for *H*≃0.88 and *L*=0 (at the green spot of the Fig. 2a) for two polarizations: 
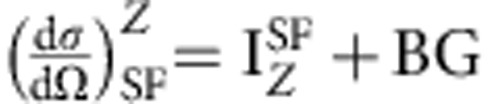
 (open symbols) and 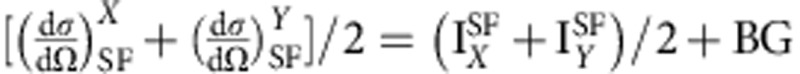 (full symbols). BG represents a spin incoherent background scattering that weakly decreases with temperature^33^. (**b**) Differential cross-sections 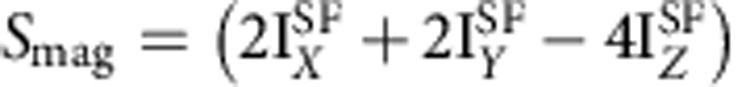 obtained from the polarization analysis around (0.88,0,0). Solid lines are guides to the eye. Intensity error bars are statistical error bars calculated by the square root of the measured intensity.

Further, one observes a diffuse magnetic scattering at wave vectors away from the magnetic rod in addition to the correlated magnetic intensity occuring along (1,0,*L*). That diffuse scattering is at least one order of magnitude weaker. Figure 4a shows the SF intensities for two polarizations around *H*=0.88 and *L*=0 (see Fig. 2c for the momentum location). On cooling, these diffuse SF intensities decrease. This is consistent with a shift of magnetic scattering towards *H*=1 (Fig. 3a), likely owing to a smooth increase of the magnetic correlation length on cooling.

We applied the standard D7 polarization analysis relation for paramagnetic systems, typically valid for disordered magnetism^33^ to get the differential cross-section, 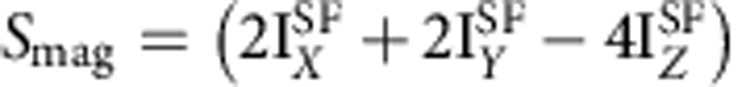 from these diffuse SF intensities. That quantity, *S*_mag_, does not depend on the background level, assuming that the background is independant of the polarization. Interestingly, *S*_mag_ exhibits a net maximum as a function of temperature at *T*_mag_=200 K (Fig. 4b). Such a cusp shape is generally characteristic of critical-like scattering around an ordering temperature, although here this does not apply fully as the correlations lengths remain finite. Acordingly, at wave vector (*H*,0,0) far away from *H*=1 (say *H*<0.8), the cusp shape disappears. Looking back to the SF intensities, the cusp can be nearly seen in the (*X*+*Y*)/2 channel although it does not occur for the *Z* polarization (Fig. 4a). The fact that the cusp is more prominent in *S*_mag_ underlines different thermal dependencies of the magnetic components of the fluctuating moments (Supplementary Note 5).

## Discussion

IUC magnetic correlations are still sizeable around optimal doping below *T*_mag_ ∼200 K in YBa_2_Cu_3_O_6.85_ (*T*_c_=89 K, *p*=0.15). Compared with samples with a lower hole doping level, the measured intensity at the Bragg position is nevertheless strongly reduced. More precisely, the magnetic intensity in YBa_2_Cu_3_O_6.85_ is ∼4 times weaker (Fig. 1d) than previous measurements performed on YBa_2_Cu_3_O_6.6_ at **Q**=(1,0,0) (ref. 11) and **Q**=(1,0,1) (ref. 5). Following the same hypotheses as before to determine the magnetic moment^9^ and keeping in mind that the magnetic intensity is proportional to *M*^2^, the magnetic moment would then be estimated to be only ∼0.05*μ*_B_ compared with 0.1*μ*_*B*_ for more underdoped YBCO^5^,^9^. In Bi_2_Sr_2_CaCu_2_O_8+*δ*_ compounds, the magnetic intensity similarly decreases rapidly from the underdoped to optimally doped samples^14^. In this study, we have established that the magnetic intensity is proportional to *T*_mag_ in the underdoped regime, whereas this scaling suddenly breaks down on approaching optimal doping.

We suggested that this effect could be triggered by a redistribution of the magnetic scattering in momentum space, namely, a shortening of the magnetic correlation length on increasing hole doping. As one observes short-range magnetic correlations in YBa_2_Cu_3_O_6.85_, one can test that scenario here considering the observed correlation lengths, *ξ*_*a*,*c*_, at 100 K. To perform that comparison, one needs to compare *ξ*_*a*,*c*_ with the resolution fonction of the 4F1 spectrometer where we have measured both YBCO samples. With the broad resolution along *H* of the spectrometer 4F1 (see Methods), the observed in-plane correlations length, *ξ*_*a*_ ∼20*a*, is too large to broaden the in-plane *H*-width and does not affect the peak intensity. In contrast, the large *L*-width along c*, Δ*L*=0.65±0.05 r.l.u., is ∼3 times broader than the resolution peak width along c*. At lower doping, the peak was found to be resolution limited along c* in YBa_2_Cu_3_O_6.6_ (ref. 11). Therefore, the *L*-integrated intensity is consistent with the ratio of *T*_mag_ measured for the two different dopings. The broad *L*-dependence can explain the sharp decrease of the peaked magnetic intensity versus doping^14^.

The observation of short-range magnetic correlations may also explain why the observation of the IUC magnetic order has not yet been corroborated by magnetic local probe measurements^9^, such as nuclear magnetic resonance^18^,^19^, owing to their much longer timescales. Indeed, finite magnetic correlation lengths could be bound to the existence of slowly fluctuating domains, with a characteristic timescale proportional to the square of the correlation length^20^. In such a line of thought, the correlation length ought to stay finite even at lower doping to explain the absence of a magnetic signal in local probes. However, one cannot give a value of the timescale from our measurement. It can only be longer than our neutron resolution timescale (∼10^−11^ s; ref. 5).

Our results show finite magnetic correlation lengths, this can be due to disorder in our sample. It is important to remember that the IUC order is stable in the presence of disorder as magnetic intensity at the Bragg peak position has been found near optimal doping in Bi_2_Sr_2_CaCu_2_O_8+*δ*_ (ref. 8), where scanneling tunnel microscopy studies^34^ report a significant disorder related to oxygen. Chain fragments in the CuO chains of YBCO can induce such a disorder in the neighbouring CuO_2_ planes that could play a role in the proliferation of magnetic domains. Alternatively, one cannot exclude that the domain formation could also be the result of a competition with another electronic instability.

In terms of the phase diagram, it is also worth pointing out that X-ray measurements recently revealed CDW order^2^,^35^, which appears systematically at lower temperature *T*_CDW_<*T*_mag_ for all doping and also for different cuprates^36^. Further, the correlation lengths of the CDW state in YBa_2_Cu_3_O_6.85_ is *ξ*_CDW_ ∼8*a*<*ξ*_*a*_ ∼20*a* weaker than the correlation lengths of the IUC order. That suggests the pre-eminence of the IUC order over the CDW instability. Disorder has been invoked also to explain the short-range CDW correlations^19^.

We report the first signature of a decoupling of the magnetic moment components as they exhibit noticeably distinct temperature dependences. The component along c* correlates first at high temperature, whereas the in-plane component is absent. Interestingly, that reveals a strong Ising character as it is expected in the original LC model^9^,^21^. In contrast, the correlated in-plane component develops only below *T*_mag_ giving rise to an apparent tilt of the magnetic moment. Most theories that are interpretating the origin of the tilt, *θ*, focus on the Bragg peak *L*=1 (refs 24, 28, 29). Our results challenge them as it remains ∼40° whatever the *L* value. It actually does not favour models in which *θ* goes to zero at *L*=0, such as LC models that include apical oxygens as an explanation for the tilt^23^,^24^, where one would expect a *L*-dependent tilt. More generally, the observed temperature dependence of *θ* is not consistent with theories where the tilt direction is due to rigid geometrical factors. The same conclusion holds when the in-plane component is related to spin orbit^22^. In contrast, in the LC approach^25^, where the planar magnetic component arises from quantum superposition of LC patterns, an Ising-like response could be recovered at high temperature once thermal fluctuations would overcome quantum effects.

Our study highlights the existence of short-range IUC magnetic correlations at very high temperature with an Ising anisotropy consistent with orbital magnetism produced by LCs. Meanwhile, the mysterious in-plane IUC correlations only develop around *T*_mag_, which matches the pseudo-gap temperature. Concommitently, the magnetic response undergoes a 2D to 3D crossover. Our study further suggests that it may exist a close connection between the pseudo-gap and the balance between in-plane and out-of-plane IUC magnetic correlations.

## Methods

### Polarized neutron diffraction

The polarized neutron experiments have been performed on two spectrometers: the triple axis spectrometer 4F1 (Orphée, Saclay; Supplementary Note 1) and the multidetector diffractometer D7 (ILL, Grenoble; Supplementary Note 2). For both, a polarizing super-mirror (bender) and a Mezei flipper are inserted on the incoming neutron beam to select neutrons with a given spin. In addition, a filter (pyrolytic graphite for 4F1 or beryllium for D7) is put before the bender to remove high harmonics. After the sample, the final polarization, *P*, is analysed by an Heusler analyser on 4F1, whereas D7 is equipped with an array of polarizing benders in front of the detectors. For each wave vector **Q**, the scattered intensity is measured in both SF and NSF channels. For the measurements on 4F1, the incident and final neutron wave vector are set to *k*_I_=*k*_F_=2.57 Å^−1^. On the diffractometer D7, the incident wave vector is taken as *k*_I_=1.29 Å^−1^. As we are using cold neutrons, we had ∼4 times better *H*-resolution compared with4F1, where Δ*H*_res_≃0.08 r.l.u. with a similar *L*-resolution Δ*L*_res_∼0.2 r.l.u., as this quantity is mostly related to the sample mosaicity. Following previous studies^5^,^6^,^8^,^11^,^12^,^14^, the search for magnetic order in the pseudo-gap phase is performed around Bragg reflections **Q**=(1,0,*L*) with integer *L* values. The sample was then aligned in the scattering plane (1,0,0)/(0,0,1), thusonly wave vectors spanning the (*H*,0,*L*) plane are accessible. D7 is equipped with a fixed *XYZ* polarization mode^33^ with *Z* being vertical and *X* and *Y* are pointing along arbitrary in-plane directions. For all of the three polarizations *X*, *Y* and *Z*, the SF and NSF channels were systematically measured over a few rocking angles of the sample to map the magnetic scattering. We then performed data reduction adapting the standard procedure^33^ and forced the magnetic scattering to be featureless at 300 K for any polarization direction. This was made to find evidence for small magnetic intensities. The procedure includes background subtraction, flipping ratio and vanadium corrections. Further, the conversion in absolute units has been performed using the Bragg peak **Q**=(1,0,0) intensity (see Supplementary Note 2 for details).

## Additional information

**How to cite this article:** Mangin-Thro, L. *et al.* Intra-unit-cell magnetic correlations near optimal doping in YBa_2_Cu_3_O_6.85_. *Nat. Commun.* 6:7705 doi: 10.1038/ncomms8705 (2015).

## Supplementary Material

Supplementary Information Supplementary Figure 1, Supplementary Notes 1-5 and Supplementary References
