# Supplementary material for: Intra-unit-cell magnetic correlations near optimal doping in YBa2Cu3O6.85
Source: Nat Commun. 2015 Jul 3;6:7705. doi: 10.1038/ncomms8705 (PMC4506545; doi:10.1038/ncomms8705)
Supplement: Supplementary Information — Supplementary Figure 1, Supplementary Notes 1-5 and Supplementary References [file ncomms8705-s1.pdf]

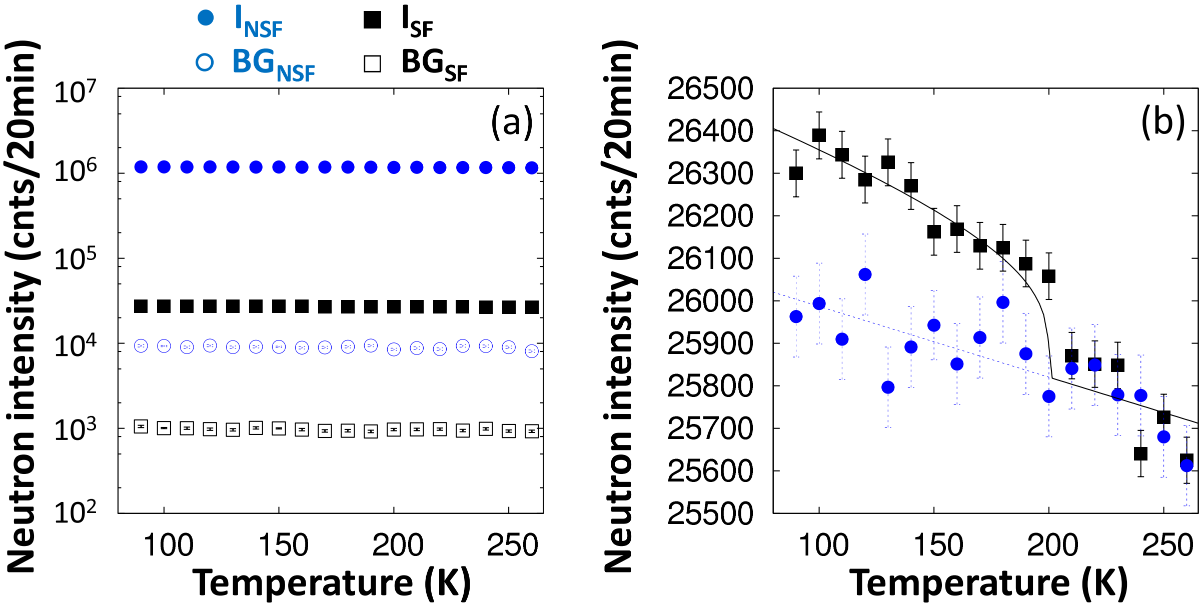

Supplementary Figure 1: (color online) (a) Temperature dependencies of the raw neutron scattering intensities measured at the Bragg peak **Q** = (1,0,1) (full symbols) and at **Q** = (0.9,0,1) (empty symbols), the background position, in the NSF channel (blue) and in the SF channel (black). (b) Temperature dependencies of intrinsic Bragg scattering (background subtracted) at **Q** = (1,0,1) in the SF channel (black) and in the NSF channel (blue) divided by a T-dependent bare flipping ratio FR°(T), so that at high temperature there is no magnetic signal. Both pictures show measurements in the **P** ∥ **Q** configuration.

Supplementary Note 1

Polarized neutron experiment on 4F1:

We here give additional information necessary for the analysis of the polarized neutron data. We report polarized neutron measurements on a $YBa_2Cu_3O_{6.85}$ (T_c = 89K) single crystal of mass ∼ 9.5 g. The polarized neutron experiments have been performed on two spectrometers: the triple axis spectrometer 4F1 (Laboratoire Léon Brillouin, Orphée, Saclay) and the diffuse scattering spectrometer D7 (Institut Laue Langevin, Grenoble).

Many details concerning neutron polarization and flipping ratio on 4F1 have been already discussed in Refs. [1–9] in the context of the magnetic intra-unit-cell (IUC) in high temperature superconducting cuprates. On 4F1, the incident and final neutron wave vector are set to $k_I = k_F = 2.57Å^{-1}$ and the energy resolution is ∼ 1 meV.

Flipping ratio:

The intra-unit-cell magnetic order produces a signal at the same position as the nuclear Bragg reflections [4, 5]. The magnetic signal is much weaker than the nuclear signal but it acts to flip the neutron spin. As polarized neutron measurements are able to distinguish nuclear and magnetic scattering, they are a very elegant tool to observe the IUC magnetic order. The scattered intensity at a given wave vector **Q** is then measured in both spin-flip (SF) and non-spin-flip (NSF) channels. The flipping ratio, FR = I_{NSF}/I_{SF}, defines the quality of the polarization. A typical flipping ratio is of the order of 40 for 4F1. Furthermore, polarized neutron scattering allows the separation of the spin orientation along three orthogonal directions. For the configuration where the polarization **P** is parallel to **Q**, the magnetic scattering is exclusively in the SF channel [4]. Consequently, we essentially carried out measurements with that polarization direction on 4F1.

We show on Supplementary Fig. 1 the results from the spectrometer 4F1 as a function of the temperature in both SF and NSF channels for the neutron polarization **P**//**Q**. To separate the magnetic scattering, it is convenient to estimate the inverse of the flipping ratio, which is given by:

$$1/FR(T) = I_{SF}/I_{NSF} = 1/FR^0(T) + I_{mag}/I_{NSF} \quad (1)$$

$1/FR^0$ represents the polarization leakage (from the NSF channel into the SF channel), characterizing the neutron beam polarization quality of the instrument. It is defined at the highest measured temperature. $1/FR-1/FR^0$ ($=I_{mag}/I_{NSF}$ where I_{mag} is the expected magnetic intensity for the IUC order) represents the normalized magnetic

intensity reported in Fig. 1. This quantity is used in order to easily compare different studies of the magnetic intensity on different samples. At the first approximation, $1/FR^0$ is supposed to be temperature independent. However, it appears empirically to be slightly temperature dependent [8]. This T-dependence is determined by measurements at Bragg peaks where the magnetic signal can be ignored such as at large $|\mathbf{Q}|$ where any magnetic signal becomes vanishingly small. In Fig. 1.a-b, the normalized magnetic intensity obtained at $\mathbf{Q}$ = (2,0,0) and $\mathbf{Q}$ = (0,0,4) is reported where a magnetic signal (if any) is beyond a threshold of detection ($< 5x10^{-5}$). We defined $1/FR^0(T)$ in our experiment as the average of the inverse of the flipping ratio at $\mathbf{Q}$ = (2,0,0) and $\mathbf{Q}$ = (0,0,4) shown in Fig. 1.a-b where no magnetic signal is observed, signifying that those two wave-vectors are good reference positions for corrections.

In order to extract the magnetic intensity, we measured the Bragg reflections $\mathbf{Q}$ = (1,0,L) (L either 0 or 1) as a function of the temperature. As the Bragg intensity appears on top of a background, we also need to measure the intensity at $\mathbf{Q}$ = (0.9,0,L) to get the temperature dependence of the background in both SF and NSF channels. Supplementary Fig. 1.a shows the temperature dependence of the SF and NSF scattering for $\mathbf{Q}$ = (1,0,1) and $\mathbf{Q}$ = (0.9,0,1). Compared to Bragg reflection, the intensity at $\mathbf{Q}$ = (0.9,0,L) is small. That background was then subtracted from the signal at $\mathbf{Q}$ = (1,0,L) to keep only the intrinsic temperature dependence of the Bragg peak. Supplementary Fig. 1.b shows the temperature dependence of the SF and NSF scattering normalized to the highest measured temperature $\sim$ 260K after the background has been removed. The NSF signal has been further divided by the T-dependent $1/FR^0(T)$. One can observe an enhancement in the SF channel corresponding to the appearance of the magnetic signal below $\sim$ 200K on top of NSF signal normalized by $1/FR^0(T)$.

Once $1/FR - 1/FR^o = I_{mag}/I_{NSF}$ is determined one can convert I_{mag} to absolute units following a method described in previous studies[1, 4]. For $\mathbf{Q}$ = (0,0,4), the square of the structure factor corresponds to $\sim$ 7 barns [1]. All cross-sections are given per formula unit (f.u.). Then, to get the Bragg intensity, we have applied the following conversion factor: $[I_{NSF}(1,0,L) * 7/I_{NSF}(0,0,4)]$. In our present case, $I_{NSF}(1,0,0)/I_{NSF}(0,0,4) \sim 4/7$, so that, if the amplitude of the magnetic intensity for both $\mathbf{Q}$ = (1,0,L) is $\sim 3x10^{-4}$ of the nuclear intensity, for $\mathbf{Q}$ = (1,0,0) the magnetic intensity is of 1.2 mbarn per formula unit (f.u.). Applying the same procedure for $\mathbf{Q}$ = (1,0,1), one gets $\sim$ 3 times weaker SF intensity, 0.4 mbarns per f.u.. Compared to previous measurements performed on $YBa_2Cu_3O_{6.6}$ at $\mathbf{Q}$ = (1,0,0) [6] and $\mathbf{Q}$ = (1,0,1) [1], the magnetic intensity in $YBa_2Cu_3O_{6.85}$ is $\sim$ 4 times weaker (Fig. 1.d).

Supplementary Note 2

D7 procedure:

We show results obtained on the multi-detector diffractometer D7 (Institut Laue Langevin, Grenoble). The current set-up of the cold neutron diffractometer D7 has been given in refs. [11, 12]. It makes use of a triple-blade monochromator which selects the incoming neutrons. A beryllium filter after the monochromator removes high harmonics. A supermirror polarizer and a Mezei flipper select neutrons with a given spin. D7 is equipped with a fixed XYZ polarization mode at the sample position. Small guiding magnetic fields are produced from a set of electric coils. The final polarization is then analysed by 66 supermirror benders over a wide range of scattering angles. Two detectors are located after each bender, giving a variable flipping ratio for each detector ranging from 15 to 35.

We use an incident wavelength of $\lambda_I = 4.86$Å ($\equiv k_I = 1.29$Å^{-1}), which corresponds to $E_I = 3.47$meV. Since it is a diffractometer, neutrons with different final energies around E_I are measured. The instrument uses bent mirror devices between sample and detector to analyse the polarization. The devices have a maximum neutron energy cut-off of $\sim$ 25 meV, meaning that D7 integrates the magnetic fluctuations from an energy range between -20 meV to E_I.

Both 4F1 and D7 allow the polarization to be arbitrarily oriented. On 4F1, as already described in previous papers [1–9], the polarization directions can be adapted to any direction relative to $\mathbf{Q}$. Contrary to the 4F1 set-up, the directions of the polarization on D7 are along arbitrary directions unrelated to $\mathbf{Q}$. The polarizations X and Y are in the scattering plane whereas Z is perpendicular to the plane thus always perpendicular to $\mathbf{Q}$. The direction X on D7 is along a direction making an angle 24° from $\mathbf{k_i}$ [12]. Measurements were performed in both SF and NSF channels, for the three X, Y, Z polarizations. We used the Lamp software provided by ILL to analyse the data (see http://www.ill.eu/instruments-support/computing-for-science/cs-software/all-software/lamp/). We applied the standard procedure which consists in a background subtraction, a flipping ratio correction and a vanadium normalization to get intensities in absolute units for each channels.

We carried out measurements of the background coming from an empty sample holder so that we could compare measurements with and without the sample. A quartz rod of the size of our $YBa_2Cu_3O_{6.85}$ sample was put in the cryostat in order to get the flipping ratio correction. The diffuse scattering from amorphous quartz is entirely nuclear. Consequently, it indicates the ratio of the correctly attributed non-spin-flip scattering cross section to the wrongly attributed spin-flip scattering cross section over all the detectors (132 detectors). This gives the flipping ratio FR for

each detector. In the limit of large FR [11]:

$$I_{SF}^{corr} = I_{SF} - \frac{1}{FR-1}(I_{NSF} - I_{SF}) \quad (2)$$

$$I_{NSF}^{corr} = I_{NSF} + \frac{1}{FR-1}(I_{NSF} - I_{SF}) \quad (3)$$

We applied the flipping ratio correction (as Eqs. 2 and 3) on our dataset in YBCO using flipping ratios determined using the quartz sample. Actually, that correction is insufficient. Indeed, the amorphous quartz sample gives broad diffuse scattering and the flipping ratios are generally reliable for correcting diffuse scattering. However, systematic errors can occur when measuring different scattering, such as an intense Bragg peak which naturally is better collimated and samples a different weighted average of the integrated polarization efficiency of the instrument. As a result, we actually observed larger flipping ratio on our YBCO single crystal than on the quartz reference. To improve the analysis, we made the assumption that no magnetic signal is present at 300K. We then adjusted the flipping ratio for each detector so that the scan is featureless at 300K for each **Q**=(1,0,L) and for each polarization. We then applied the flipping ratio correction of Eq. 2 with a corrected set of flipping ratios. The intrinsic magnetic signal appears then clearly at 100K on top of the flat scan at 300K (Fig. 2.c). Finally, we performed measurements on a vanadium rod, to correct the data for detector efficiency and to be able to calculate the cross section in absolute units. Further, following the procedure discussed above for 4F1, the conversion in absolute units has been cross-checked using the Bragg peak **Q**=(1,0,0) intensity.

The measurements reported are mostly for the trajectory crossing L=0.25 for H=1 which is where the contrast between the magnetic scattering over the nuclear scattering is the best: on the one hand, the nuclear scattering from the Bragg peak at L=0 is about 10 times weaker at L=0.25 and, on the other hand, the magnetic scattering is not much reduced. As shown by the correlations along c* (Fig. 3.b), magnetic intensity at L=0.25 belongs to the same peak as for L=0. The reported q-widths in the text correspond to the projection along H of the detector trajectory (redline on Fig. 2.a). This projection assumes that the L-dependence of the magnetic scattering is very broad, as this is what found in the measurements (Fig. 3.b).

Supplementary Note 3

Averaging intensities:

In order to get better contrast of the intensities for particular wave-vectors, we averaged intensities $\mathrm{I_i}$ from N detectors using $\bar{\mathrm{I}} = \sum_{\mathrm{i=1}}^{\mathrm{N}} \mathrm{I_i/N}$. That quantity is actually related to the integrated area of the peak. The intensity at each detector $\mathrm{I_i}$ (*i.e.* at a wave vector q_i) is a sum of a background BG_i and a contribution coming from a peak centered at q_0 with a FWHM of Δq and a magnetic amplitude $\mathrm{I_0}$. For a Gaussian description of the peak, $\mathrm{I_i}$ is written as

$$I_i = BG_i + I_0 \exp(-4\ln 2 (q_i - q_0)^2/\Delta q^2) \quad (4)$$

The averaged intensity $\bar{\mathrm{I}}$ can be written as,

$$\overline{I} = \sum_{i=1}^{N} I_i/N = \overline{BG} + I_0 \frac{\int_{q_1}^{q_N} \exp(-4\ln 2(x-x_0)^2/\Delta q^2)}{\int_{q_1}^{q_N} dq} = \overline{BG} + I_0 \frac{\Delta q}{q_N - q_1}\sqrt{\frac{\pi}{4\ln 2}} \quad (5)$$

$(\mathrm{q_N} - \mathrm{q_1})$ is the width on which we are averaging intensities, $\overline{\mathrm{BG}}$ is the averaged background level taken at high temperature near the peak. $I_{av} = \overline{I} - \overline{BG}$ is the quantity plotted in Fig. 3 of the manuscript for N=10, it corresponds to an integration in a q-range of $|q_N - q_1| \simeq 0.09$ r.l.u. along H. I_{av} is proportional to the product of the intensity at the maximum and of the q-width: $\propto \mathrm{I_0 \Delta q}$. As $\Delta q \propto 1/\xi_a$, $\overline{I}$ then typically measures the ratio of the magnetic intenisity to the in-plane correlation length: $\overline{I} \propto I_0/\xi_a$.

Supplementary Note 4

Neutron cross-sections:

For the case of a magnetic state with an anisotropic magnetic moment pointing along a generic direction $\mathrm{M} = (\mathrm{M_a}, \mathrm{M_b}, \mathrm{M_c})$, one can estimate the magnetic neutron cross-sections for each neutron polarization I_α with $\alpha = (\mathrm{X}, \mathrm{Y}, \mathrm{Z})$. For a twinned sample, both directions (1,0) and (0,1) are equivalent and one can define $\mathrm{M_{ab}} = \sqrt{\mathrm{M_a^2} + \mathrm{M_b^2}}$ as the in-plane component and $\mathrm{M_c}$ as the out-of-plane component[4]. The neutron intensity for any arbitrary direction of the polarization $\alpha = (\mathrm{X}, \mathrm{Y}, \mathrm{Z})$ can be written as [11], $(\frac{d\sigma}{d\Omega})^\alpha_{\mathrm{sf}} = \mathrm{I}^{\mathrm{SF}}_\alpha + \mathrm{BG}_\alpha$ where BG_α is the background. For a XYZ-polarization defined by an arbitrary angle β between X and **Q**, $\mathrm{I}^{\mathrm{SF}}_\alpha$ can be written as,

$$I_Z^{SF} \propto (1 - q_l^2)M_c^2 + \frac{q_l^2}{2}M_{ab}^2 \sim M_c^2 \tag{6}$$

$$I_Y^{SF} \propto \frac{1}{2}M_{ab}^2 + I_Z^{SF}\ sin^2\beta \tag{7}$$

$$I_X^{SF} \propto \frac{1}{2}M_{ab}^2 + I_Z^{SF}\ cos^2\beta \tag{8}$$

where $\mathrm{q_l} = \frac{2\pi}{\mathrm{c}}\mathrm{L}/|\mathbf{Q}|$ tends to zero in our study for L$\sim$ 0. The same formulas can be applied for the 4F1 setup but with $\beta = 0$, the polarization along X for 4F1 being along **Q**.

Supplementary Note 5

Diffuse scattering:

With detectors covering a wide momentum space over a couple of Brillouin zones, D7 is nominally a spectrometer designed to measure diffuse scattering that is broad in Q-space at positions away from the Bragg position. To analyze the measured spin-flip intensities, one takes advantage of the polarization analysis to extract the magnetic scattering from the background. The standard D7 full XYZ polarization analysis separates the (para)magnetic intensity independently from the background (such as nuclear and spin-incoherent scattering). The only assumption is that the background is not polarization dependent. This is readily the case at position away from the Bragg peaks. One can consider the difference of these cross-sections, $\mathrm{S_{mag}}$ (independent of the background):

$$S_{mag} = 2I_X^{SF} + 2I_Y^{SF} - 4I_Z^{SF} \tag{9}$$

$\mathrm{S_{mag}}$ is represented in Fig. 4.b of the manuscript. In the case of disordered magnetism, that cross section corresponds to paramagnetic scattering for isotropic magnetic correlations *i.e.* $\mathrm{M_a^2} = \mathrm{M_b^2} = \mathrm{M_c^2}$. This is extensively measured on D7 [11]. It is worth to note that Eq. 9 determines the full magnetic scattering only for paramagnetic scattering. In this limit, it gives $\mathrm{S_{para}} \propto \frac{2}{3}\mathrm{g^2F^2\,(Q)\,S(S+1)}$ where g is the Landé factor, F(Q) is the relevant magnetic form factor and S is the effective spin. This does not apply for anisotropic magnetic systems.

For the analysis of Fig.4, one needs to consider that using Eqs. 6-8 above: (i) $\mathrm{I_Z^{SF}}$ is dominated by the out-of-plane component of the diffuse scattering, (ii) $(\mathrm{I_X^{SF}} + \mathrm{I_Y^{SF}})/2$ is given by half of the sum of the in-plane and out-of-plane components, (iii) $\mathrm{S_{mag}}$ stands for twice the difference between the in-plane and out-of plane components. If the diffuse scattering were purely isotropic, *i.e.* $\mathrm{M_{ab}^2} = 2\ \mathrm{M_c^2}$, the three quantities reported in Fig.4 should show a unique temperature-dependence, weighted by factors 1, 3/2 and 2 respectively. From these considerations, it appears that the diffuse scattering is anisotropic and that the in-plane and out-of-plane display distinct T-dependencies, with a marked difference marked around $\mathrm{T_{mag}}$ in particular.

Supplementary references:

[1] Fauqué, B., *et al.* Magnetic Order in the Pseudogap Phase of High-Tc Superconductors. *Phys. Rev. Lett.* **96**, 197001 (2006).

[2] Li, Y. *et al.* Unusual magnetic order in the pseudogap region of the superconductor $HgBa_2CuO_{4+\delta}$. *Nature* **455**, 372-375 (2008).

[3] De Almeida-Didry, S., *et al.* Evidence for intra-unit-cell magnetic order in $Bi_2Sr_2CaCu_2O_{8+\delta}$. *Phys. Rev. B (RC)* **86**, 020504 (2012).

[4] Bourges, P. & Sidis Y. Novel magnetic order in the pseudogap state of high-Tc copper oxides superconductors. *C. R. Physique* **12**, 461-479 (2011).

[5] Sidis, Y. & Bourges, P. Evidence for Intra-Unit-Cell magnetic order in the pseudo-gap state of high-Tc cuprates. *J. Phys.: Conf. Ser.* **449** 012012 (2013).

[6] Mook, H.A., Sidis, Y., Fauqué, B., Balédent, V.& Bourges, P. Observation of magnetic order in a superconducting $YBa_2Cu_3O_{6.6}$ single crystal using polarized neutron scattering. *Phys. Rev. B* **78**, 020506 (2008).

[7] Li, Y. *et al.* Magnetic order in the pseudogap phase of $HgBa_2CuO_{4+\delta}$ studied by spin-polarized neutron diffraction. *Phys. Rev. B* **84**, 224508 (2011).

[8] Balédent, V., Haug, D., Sidis, Y., Hinkov, V., Lin, C.T. & Bourges P. Evidence for competing magnetic instabilities in underdoped $YBa_2Cu_3O_{6+x}$. *Phys. Rev. B* **83**, 104504 (2011).

[9] Mangin-Thro, L. *et al.* Characterization of the intra-unit-cell magnetic order in $Bi_2Sr_2CaCu_2O_{8+\delta}$. *Phys. Rev. B* **89**, 094523 (2014).

[10] Shekhter, A. *et al.* Bounding the pseudogap with a line of phase transitions in $YBa_2Cu_3O_{6+\delta}$. *Nature* **498**, 75-77 (2013).

[11] Stewart, J.R. *et al.* Disordered materials studied using neutron polarization analysis on the multi-detector spectrometer, D7. *J. Appl. Cryst.* **42**, 69-84 (2009).

[12] G. Ehlers, G. Stewart, J.R., Wildes, A.R., Deen, P.P. & Andersen, K.H. Generalization of the classical xyz-polarization analysis technique to out-of-plane and inelastic scattering. *Review of Scientific Instruments* **84**, 093901 (2013).
